# Posterior cervical instrumented fusion case complicated by acute generalized erythematous pustulosis

**DOI:** 10.1093/jscr/rjaf126

**Published:** 2025-03-12

**Authors:** Ali Bakhsh, Masna Inam, Sebastian Trifoi, Mohammad Saleemi, Oluwaseun Sobowale, Narendra Rath, Neil Buxton, Marcus de Matas

**Affiliations:** Department of Neurosurgery, Neurosurgery, The Walton Centre NHS Foundation Trust, Lower Lane, Liverpool L9 7LJ, United Kingdom; Department of Neurosurgery, Neurosurgery, The Walton Centre NHS Foundation Trust, Lower Lane, Liverpool L9 7LJ, United Kingdom; Department of Neurosurgery, Neurosurgery, The Walton Centre NHS Foundation Trust, Lower Lane, Liverpool L9 7LJ, United Kingdom; Department of Neurosurgery, Neurosurgery, The Walton Centre NHS Foundation Trust, Lower Lane, Liverpool L9 7LJ, United Kingdom; Department of Neurosurgery, Neurosurgery, The Walton Centre NHS Foundation Trust, Lower Lane, Liverpool L9 7LJ, United Kingdom; Department of Neurosurgery, Neurosurgery, The Walton Centre NHS Foundation Trust, Lower Lane, Liverpool L9 7LJ, United Kingdom; Department of Neurosurgery, Neurosurgery, The Walton Centre NHS Foundation Trust, Lower Lane, Liverpool L9 7LJ, United Kingdom; Department of Neurosurgery, Neurosurgery, The Walton Centre NHS Foundation Trust, Lower Lane, Liverpool L9 7LJ, United Kingdom

**Keywords:** cervical, complication, autoimmune, spine

## Abstract

This case report describes the first case of acute generalised erythematous pustulosis (AGEP) following cervical spinal surgery. A 74-year-old male post-operatively developed a painful, exudative bullous rash progressing from the posterior cervical wound site. Initial management with antibiotics for suspected cellulitis failed, leading to further investigation and a diagnosis of AGEP by dermatology. Treatment with topical steroids resulted in rapid improvement, indicating the importance of early recognition and intervention. The patient developed secondary wound infection requiring surgical debridement. AGEP is linked to IL36RN gene mutations. This case underscores the necessity for clinicians to consider AGEP in peri-operative skin reactions, emphasizing early steroid intervention and vigilant monitoring for secondary infections.

## Introduction

Acute post-operative wound changes (Day 1) in cervical spinal surgery is rare and usually indicates a non-infectious pathology such as Type I-III hypersensitivity reactions, vascular, or pressure related skin erythema. We present a case of a progressive, painful and exudative peri-wound rash caused by a rare dermatological condition, AGEP. Treatment of this condition involve topical steroids. This is the first report of this condition associated with instrumented spinal surgery in the literature to the author’s knowledge. The patient gave their written consent to publish this report.

## Case presentation

A 74-year-old male, with a background of chronic leg cellulitis, underwent an elective posterior cervical spine decompression and instrumented postero-lateral fusion of vertebrae C4 to C7 for degenerative spinal stenosis causing right sided C6-7 radiculopathy, mild myelopathy and 4/5 MRC grade power right upper limb globally. The operation and immediate recovery were unremarkable with improvement in patient’s neurology.

**Figure 1:**
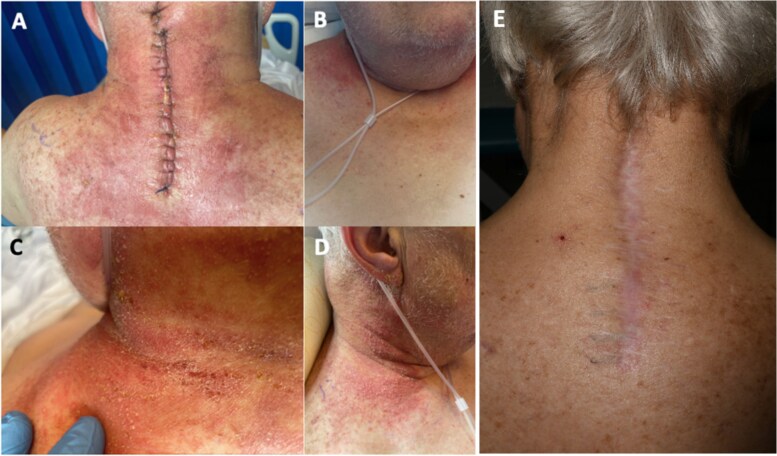
Post-operative day 2 pictures of erythematous, non-raised, posterior cervical wound (A), anterior upper chest (B), posterior neck crease with neck flexed (C), right neck showing pinpoint pustular heads (D), and 6 month follow-up cervical wound (E).

On post-operative Day 1, the patient developed a progressive, erythematous, painful, exudative bullous rash which started from the wound site (midline cervical spine), and progressed cranially towards the occiput, laterally to anterior shoulders, anterior neck and upper chest, and inferiorly to encompass the skin overlying the scapulae (Fig. 1).

On examination, the patient was uncomfortable with worsening upper back pain and tenderness on palpation; vital signs, including temperature, were within normal ranges. Laboratory investigations showed a raised CRP (C-reactive protein, 154 mg/dl; normal <1). The rash was blanching with pin-hole sized pustules. There was no associated swelling. Nikolsky sign was negative. The operative wound was involved but remained well opposed. Initial management with intravenous antibiotics for cellulitis was initiated but clinical course and CRP worsened.

On post-operative Day 3, dermatology opinion was sought and a clinical diagnosis of AGEP was made. 0.2% topical corticosteroid, oral antihistamines, skin moisturizers and analgesia were initiated. Over the next 48 hrs, the CRP improved, and the rash regressed to almost complete resolution by Day 5. A skin swab returned no growth after extended cultures.

A magnetic resonance imaging of cervical spine during this episode showed a small, superficial non-enhancing collection below the surgical wound. This was managed conservatively, and the patient discharged. Unfortunately, the patient returned 1 week later with pus egress from the wound and was taken to theatre for a wound washout and debridement. Intra-operatively, no deep collection was found and metalwork was retained. Samples grew staphylococcus epidermidis. He completed his antibiotic regime. This patient had a further hospital admission with infective endocarditis and pneumonia. At 6-month follow-up, this patient made a remarkable recovery and is mobilizing independently, normal neurology of his upper limbs, good wound healing (Fig. 1) with only mild neck pain. His pre-operative neck disability index was 72%, now 6% and pre-operative myelopathy disability index of 60%, is now 7%.

## Discussion

AGEP is a rare dermatosis with an incidence of two to three per million, that presents as multiple pustules on a generalized erythematous eruption [1, 2]. It is overwhelmingly caused by a drug reaction [3]. This patients’ only new drug was a single cephalosporin dose given on anaesthetic induction as per departmental guidelines.

About 10% of patients with AGEP have associated organ dysfunction, but secondary infection is uncommon [4]. In this case, the vicinity of a surgical wound and the pro-inflammatory, exudative tissue microenvironment made secondary wound infection more likely. AGEP is diagnosed clinically with supportive investigations including raised inflammatory markers, skin biopsy showing subcorneal pustules filled with neutrophils and patch testing to identify causative agent [1].

**Figure 2:**
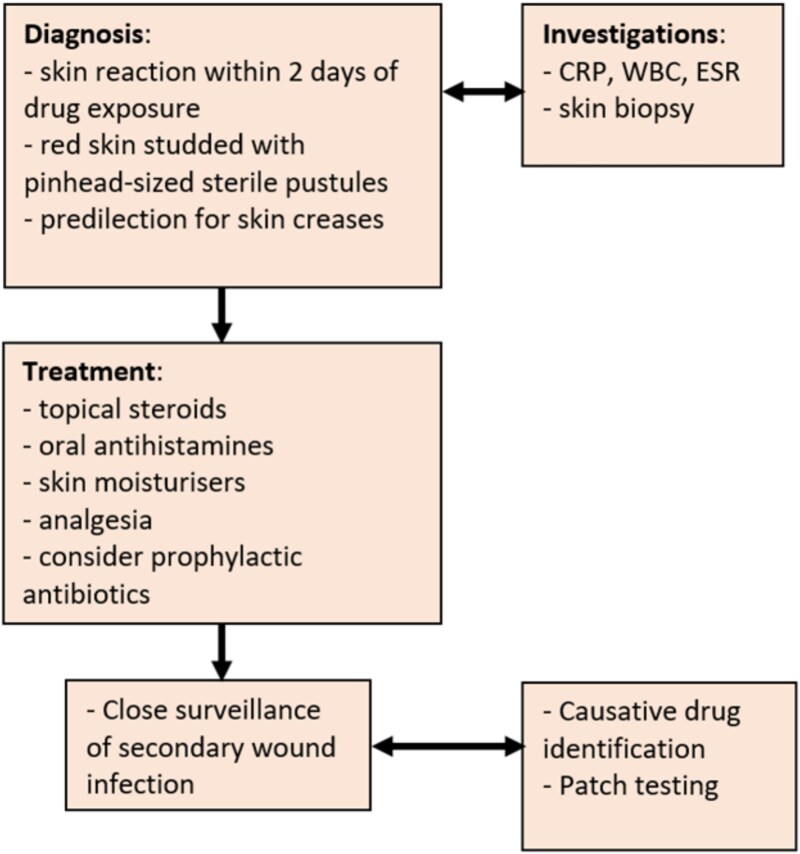
Suggested treatment pathway for diagnosis and treatment of acute generalized exanthematous pustulosis (AGEP).

Surgical teams need to consider AGEP in the differential diagnosis of a patient with acute, progressive erythema and pustules in the peri-operative context. Accurate and timely diagnosis of AGEP is vital as treatment vastly differs from infection, it can be life threatening, and there may be lifelong implications regarding drug sensitivity. A key message of this case report is the need for early use of topical steroids to treat AGEP as application of steroids to a surgical wound is usually against wound healing dogma. If suspected, we recommend a diagnostic and follow-up pathway described in Fig. 2.

This patients’ quick succession of AGEP, wound infection, endocarditis, and pneumonia may suggest an underlying susceptibility to infection. Indeed, recent research suggests that patients with AGEP are associated with homozygous IL36RN gene mutations [5,6]. These genetic abnormalities make patients more susceptible to pustulosis when prescribed certain medications or when exposed to infection. The IL36RN gene codes for interleukin 36 receptor antagonist, which is primarily found in the skin where it helps regulate inflammation by downregulating the pro-inflammatory the nuclear factor kappa B (NF-κB) and mitogen‑activated protein kinase (MAPK) pathways [7]. Dysregulation of these pathways have been implicated in attenuation of staphylococcal pneumonia [8].

## Conclusion

AGEP is a rare cause of acute post-operative skin rash and is treated with topical steroids. Clinical team should be aware of, and patient counselled, for secondary wound infection. Drug sensitivity needs to be considered and appropriate work-up may be required.
